# A clinically silent tumour of adrenal myelolipoma: A case report

**DOI:** 10.1016/j.ijscr.2020.05.056

**Published:** 2020-06-01

**Authors:** Nornazirah Azizan, Ohnmar Myint, Aye Aye Wynn, Tin Tin Thein, Firdaus Hayati, Nik Amin Sahid Nik Lah

**Affiliations:** a Department of Pathobiology and Medical Diagnostic, Faculty of Medicine and Health Sciences, Universiti Malaysia Sabah, Kota Kinabalu, Sabah, Malaysia; b Department of Surgery, Faculty of Medicine and Health Sciences, Universiti Malaysia Sabah, Kota Kinabalu, Sabah, Malaysia

**Keywords:** Benign neoplasms, CAT scan, Myelolipoma

## Abstract

**Introduction:**

Adrenal myelolipoma is a rare, non-functional, benign neoplasm which is constituted of mature haematopoietic elements and adipose tissues in various proportions. It is diagnosed accidentally and frequently with the widespread use of imaging modalities.

**Presentation of case:**

We report a 63-year-old lady with incidental findings of adrenal tumour on computed tomography (CT) scan during a routine medical check-up. She underwent tumour resection in view of a large tumour of 7 cm in size.

**Discussion:**

CT scan is sensitive to diagnose adrenal myelolipoma in view of its fat-laden property and useful to monitor the tumour progress. Even previously she opted for conservative management; the decision for surgery was made in view of enlarging tumour and risk of surrounding tissue compression.

**Conclusion:**

With increased awareness, the detection rate of this tumour is improving, hence able to prevent the complications of a large tumour such as compression, bleeding and tumour necrosis.

## 1 Introduction

Myelolipoma is a rare, non-functional, benign neoplasm which is composed of mature haematopoietic elements and adipose tissues in various proportions. It is localized in the retroperitoneum, mostly involving the adrenal glands. In the past, it was discovered at autopsy but nowadays is usually diagnosed accidentally and frequently with the widespread use of current imaging modalities. The use of ultrasonography, computed tomography (CT), and magnetic resonance imaging (MRI) has enhanced the detection rate up 15% of incidental adrenal masses [1]. The incidence of adrenal myelolipoma is 0.08–0.2% from all primary tumours of the adrenal glands [1].

Adrenal myelolipoma has an equal gender distribution and is commonly found between the 5th and 6th decades of life with a mean age of 62 years [2]. It is usually unilateral and non-secreting in nature. It is commonly smaller than 4 cm in size upon initial identification with the largest reported adrenal myelolipoma measuring 31 cm × 24.5 cm × 11.5 cm and weighing 6 kg [3]. We revisit this rare variant of lipoma of the adrenal gland that occurs in a 63-year-old female and discuss its literature review. This work has been reported in line with the SCARE criteria [4].

## 2 Case report

A 63-year-old female was noted to have an adrenal mass during her medical check-up 7 years ago. She has underlying hypertension with one anti-hypertensive drug. Otherwise, she does not have endocrine disorders or associated comorbidities in the patient and relatives. During the initial finding, it was 4.5 cm in size. Since it was asymptomatic, she was decided for routine imaging follow up. She underwent a two-yearly computed tomography (CT) scan to monitor the growth of the tumour. It remained stable initially until recently it started to grow to a size of 7 cm. The CT features were in favour of right benign adrenal tumour but in view of the risk of compressive symptoms, she was decided for surgical resection of the tumour via laparoscopic retroperitoneal approach. The surgery went well without any intraoperative and postoperative complications. She was discharged after day 2. There was no further treatment or follow up required.

**Fig. 1:**
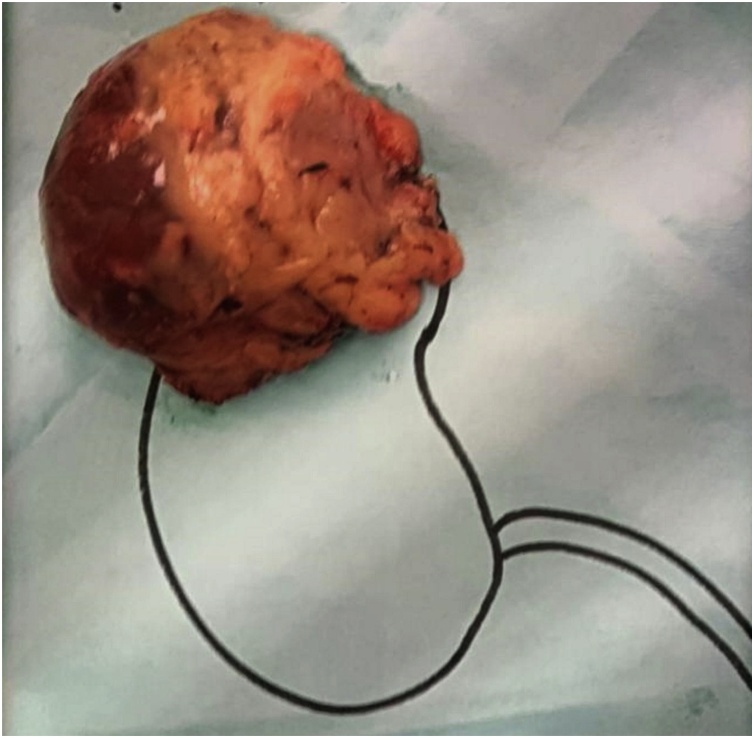
Gross features show a tumour with lobulated surface partially covered by fat tissue measuring 9 cm × 8 cm × 4 cm.

Grossly the tumour was firm with a lobulated surface measuring 95 mm × 85 mm × 40 mm and weighing 140 g (Fig. 1). It was partly covered by fatty tissue with an intact fibrous capsule. Serial sections showed a well-circumscribed tumour with heterogeneous yellowish to a greyish cut surface. Few foci of small haemorrhagic spots were seen. No necrosis identified. There is a remnant of adrenal gland attached to the lesion measuring 70 mm × 10 mm × 6 mm. Cut section of the adrenal gland is unremarkable.

**Fig. 2:**
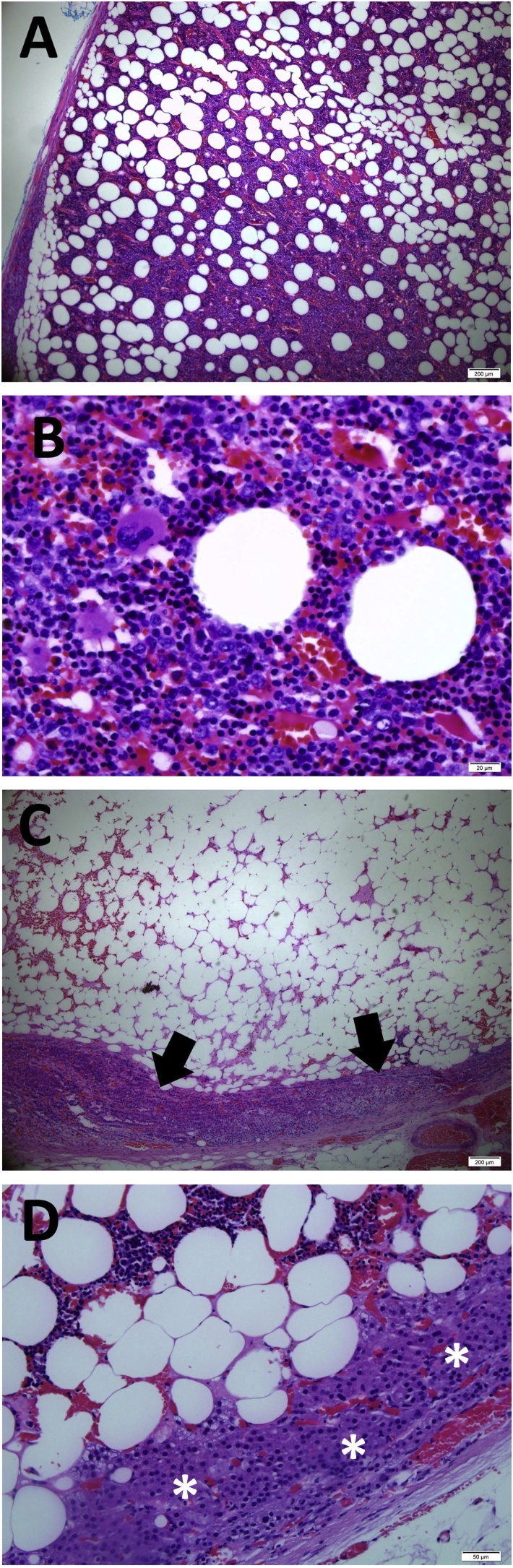
(A) Histopathologic picture showing an encapsulated tumour of myelolipoma composed of mature fat cells mixed with hematopoietic elements (×4 magnification). (B) High power view of the trilineage hematopoietic element with megakaryocytes (×40 magnification). (C) Low power view of lipomatous tumour component with attenuated adrenal cortex at the periphery (arrow) (×4 magnification). (D) Tumour with adjacent adrenal cortex (*) (×20 magnification).

Histologically, it is a well-circumscribed tumour composed of lobules of mature univacuolated adipocytes separated by fibrous septae and rimmed by a thin capsule (Fig. 2A). There were intervening blood vessels and bone marrow elements containing trilineage hematopoietic cells with an increase in the number of megakaryocytes (Fig. 2A and B). Foci of haemorrhages were seen within the adipose component. No lipoblast or atypical stromal cell seen. No necrosis or calcification identified. There was an attenuated adrenal cortical tissue seen at the periphery (Fig. 2C and D). No evidence of malignancy seen. The features are in keeping with myelolipoma.

## 3 Discussion

Adrenal myelolipoma is a very rare variant of adrenal lipoma. In our case, she is 63 years of age, which falls to the mean age group and the tumour size is 7 cm which is larger than the baseline of 4 cm. It is usually asymptomatic, but it can cause flank pain and abdominal discomfort from compression to the surrounding structures, tumour necrosis, rupture, or bleeding leading to haemorrhagic shock [2]. Due to the tumour size of 7 cm and risk of local compression, our patient was counselled for a surgical resection.

Routine imaging modalities such as ultrasonography, CT, and MRI are all effective in diagnosing adrenal myelolipoma. Among all, CT is the most sensitive modality on the basis of the fat-laden tumour [2]. Since it is non-functional, biochemical evaluation especially on the endocrinology may not be useful, although there is a report of a hormone-secreting myelolipoma causing hypertension [1,5]. Huge sized tumour in CT imaging warrants a big concern for malignancy namely liposarcoma, a fat-containing adrenocortical carcinoma (ACC), and adrenal teratoma. However, among benign differential diagnoses in CT scan should include renal angiomyolipoma and retroperitoneal lipoma.

In cases when the diagnosis can not be made radiologically or in any doubt of malignant potential, the use of fine needle aspiration cytology (FNAC) can be considered [2]. FNAC might give a clue of a lipomatous tumour and be able to detect atypical cells if there is a presence of atypical stromal cells. This will help in the management plan. However, in this case, FNAC is not required as this is a slow-growing tumour with no suspicious features of malignancy radiologically.

Histological assessment can be made on the resected specimen. Thorough gross and microscopic evaluation is needed for confirmatory diagnosis and to exclude malignancy. Specimens should be sampled 1 cm apart for microscopic analysis in order not to miss any malignancy. Microscopically, a typical myelolipoma showed the presence of a mixture of mature adipocytes with foci of trilineage hematopoietic elements [2].

Management of adrenal myelolipoma should be per individual basis. In a lesion of less than 4 cm in size, conservative treatment can be applied with an imaging technique. Surgery is indicated in symptomatic tumours, rapidly growing lesions, or tumours more than 6 cm in size [1]. This is to avoid the risk of abdominal pain or life-threatening rupture and haemorrhage. The laparoscopic approach is more superior to the open method as it can lead to lower morbidity especially on surgical site infection and lung complications, faster recovery and hospital discharge. However, laparoscopic adrenalectomy is not warranted for masses larger than 10 cm or with adhesions and infiltration of the surrounding structures [1]. The prognosis of adrenal myelolipoma is remarkable with recurrence-free survival rates of up to 12 years [1].

## 4 Conclusion

Adrenal myelolipomas are clinically silent tumours and mainly benign in nature. Patients with adrenal myelolipoma are usually presented with incidental findings. This type of tumour can be definitively diagnosed with CT imaging. With increased awareness, the detection rate of this tumour is increasing, hence able to prevent the complications of a large tumour.

## Declaration of Competing Interest

The authors have no conflict of interests to declare.

## Sources of funding

The study did not receive any funding.

## Ethical approval

No ethical clearance required as it only involves case report.

## Consent

Written informed consent was obtained from the patient.

## Author contribution

Case design and writing – NA, FH.

Case collection – OM.

Literature review – AAW, TTT, NASNL.

Acquisition of histology figures – NA.

Manuscript editing – FH.

## Registration of research studies

No ethical clearance required as it only involves case report.

## Guarantor

Firdaus Hayati.

## Provenance and peer review

Not commissioned, externally peer-reviewed.
